# COSIBAS Platform—Cognitive Services for IoT-Based Scenarios: Application in P2P Networks for Energy Exchange

**DOI:** 10.3390/s23020982

**Published:** 2023-01-14

**Authors:** Diego Gutiérrez Martín, Sebastian Lopez Florez, Alfonso González-Briones, Juan M. Corchado

**Affiliations:** 1 Air Institute, IoT Digital Innovation Hub (Spain), Carbajosa de la Sagrada, 37188 Salamanca, Spain; 2 Grupo de Investigación BISITE, Departamento de Informática y Automática, Facultad de Ciencias, Instituto de Investigación Biomédica de Salamanca, Universidad de Salamanca, Calle Espejo 2, 24.2, 37007 Salamanca, Spain; 3 Department of Electronics, Information and Communication, Faculty of Engineering, Osaka Institute of Technology, Osaka 535-8585, Japan; 4 Pusat Komputeran dan Informatik, Universiti Malaysia Kelantan, Karung Berkunci 36, Pengkaan Chepa, Kota Bharu 16100, Malaysia

**Keywords:** cognitive services, cognitive platform, energy negotiation, smart cities sustainable cities, machine learning

## Abstract

The revolution generated by the Internet of Things (IoT) has radically changed the world; countless objects with remote sensing, actuation, analysis and sharing capabilities are interconnected over heterogeneous communication networks. Consequently, all of today’s devices can connect to the internet and can provide valuable information for decision making. However, the data collected by different devices are in different formats, which makes it necessary to develop a solution that integrates comprehensive semantic tools to represent, integrate and acquire knowledge, which is a major challenge for IoT environments. The proposed solution addresses this challenge by using IoT semantic data to reason about actionable knowledge, combining next-generation semantic technologies and artificial intelligence through a set of cognitive components that enables easy interoperability and integration for both legacy systems and emerging technologies, such as IoT, to generate business value in terms of faster analytics and improved decision making. Thus, combining IoT environments with cognitive artificial intelligence services, COSIBAS builds an abstraction layer between existing platforms for IoT and AI technologies to enable cognitive solutions and increase interoperability across multiple domains. The resulting low-cost cross platform supports scalability and the evolution of large-scale heterogeneous systems and allows the modernization of legacy infrastructures with cognitive tools and communication mechanisms while reusing assets.

## 1. Introduction

COSIBAS seeks to take the next step in IoT-based applications and solutions, integrating context-aware computing [1] to address a new challenge regarding incompatibility between devices. Semantics has been a key enabler towards a complete and general description of connected objects by removing relational ambiguities and improving context awareness [2,3]. Semantic descriptions that relate an object in a virtual form lead to innate aggregate capabilities in physical devices, which facilitates dynamic process updating. The contextual information present in physical devices is not necessarily static in time; thus, devices require semantic richness with more contextual details as they interact with applications and other reasoning engines [4,5]. The constant evolution of IoT has resulted in a certain level of complexity due to the large number of heterogeneous object implementations, sensing data and suggested services [6].

Therefore, objects from different manufacturers are connected to each other, and the generated data have different encoding formats leading to a complex data exchange task, including semantic heterogeneity [7]. The variety of these objects and their constantly changing requirements and deployment contexts further complicate their management and configuration tasks. These challenges arise due to the absence of a unified and standardized model for IoT devices along with their data and services. Therefore, semantic concepts play a key role in IoT due to their efficiency in addressing issues of heterogeneity [8,9], interoperability and data interpretation [10].

The establishment of knowledge-level interoperability between data from other fields, such as climatic analysis, has recently made significant strides, according to researchers in the field of statistical web technology [11,12]. According to the studies, ontology is essential to the structure of the semantic web since it serves as a specialized language for modeling domains shared by heterogeneous entities [13]. The addition of semantics to the data sent between the parties enables a clear understanding of the knowledge shared by both parties, which improves the effectiveness of data exchange by removing misunderstandings [14,15].

In this study, we propose a solution to these problems, which consists of creating heterogeneous data sources and analyzing them using AI algorithms to increase the overall efficiency of the target system, which will consider the devices and services currently on the market by adapting the intelligence layer, making use of semantics to support inter-object communications and without forcing other solutions to adapt. The use of these technologies makes it possible to strategically explore the semantic relationships between the different energy devices in a network, allowing knowledge to be spread throughout the network and easily recognized by the end users.

At the same time, this allows simulating the processes of a P2P energy trading system within a smart city in order to optimize the transfer of energy between producers and consumers. Cognitive services will be able to interpret and evaluate the state of the entire system with the aim of building and performing transactions in a cognitive model, which is the basis for interaction, decision-making and support for intelligent mechanisms that enable seamless interoperability.

In summary, the novel contributions of this work include the development of a semantic system that can simulate what the energy negotiation process would look like in a real environment using data from external energy services. A case study examining the results of the work packages will be tested in a model that allows for the simulation of these processes so that the feasibility of these hypotheses can be demonstrated using real data in a simulated environment.

The remainder of this article is structured as follows: Section 2 presents the relevant literature, including comparisons between different methods as well as adoptions of outside ideas. Section 3 contains the architecture, including a description of the architecture in detail. Section 4 focuses on the complete workflow proposed for the machine-learning technique for solar-power-generation prediction. Section 5 discusses the machine-learning problem for wind-power-generation prediction. Section 6 describes the trading stage, and lastly, in Section 7, we summarize our work and outline possible future work.

## 2. Related Work

A general description is provided of the most recent IoT applications in semantic representation approaches. First, we study the proposed approaches in the IoT in general. Second, we are interested in approaches that define the semantics of IoT-based systems; therefore, in this section, we indicate recent survey papers on these topics.

The first study in this research area was published in 2012 by Barnaghi et al. [3] who explained the importance of defining and presenting IoT semantics to resolve the heterogeneity and ambiguity of the large amount of data collected through connected objects and to ensure interoperability between IoT systems. From this perspective, the authors present an overview of some existing ontologies designed to represent sensors and their data, such as the O&M and SSN ontologies.

In [16], an overview of SWT used in different layers of IoT systems is emphasized, along with important ontologies for developing IoT applications and services—namely, SSN [17], IoT Ontology [18] and IoT-O [19]. Following this dynamic, many IoT ontologies have been proposed by researchers with the goal of achieving semantic interoperability between heterogeneous IoT devices. LOV4IoT [20] provides a catalog of over 400+ ontologies spanning different domains, such as IoT, WoT, transportation, health, weather and food. Of these, approximately 27+ ontologies were developed explicitly to address IoT interoperability. However, they do not follow Semantic Web best practices, making them difficult for developers to adopt.

A notable exception is the W3C SSN ontology [17], which was jointly developed by several research organizations and became the W3C standard ontology for semantic sensor networks in 2017. From this, around 24 IoT ontologies were derived, which referenced the SSN concept showing its wide acceptance and usage. SAREF is a smart device reference ontology developed with the support of the European Commission [21]. It provides modular building blocks for representing devices in a smart home environment, such as lists of functions, commands and states that can be combined to create complex functions in a single device. Recently, a new field of research, the "Internet of Things" (WoT), has begun to connect internet-connected objects (ICOs) to networks, allowing for transparent access to data.

Girard et al. [22] clearly mentioned in their study that IoT itself is not sufficient to solve the semantic interoperability problem and needs the help of ontologies to conceptualize it better. The Semantic Web of Things (SWoT) [23,24] is the most recent field of research that aims to combine the power of Semantic Web and WoT technologies to achieve interoperability [25,26,27,28].

## 3. Architecture

The COSIBAS design is built on the fundamental FIWARE architecture for IoT, to which the COSIBAS platform has added additional generic components. This section displays many architectural perspectives using Kruchten’s 4 + 1 model, or "quote Kruchten." The "Recommended Practice for Architecture description of Software-Intensive Systems" standard published by IEEE 15 and used to describe the architecture from many angles is compatible with this paradigm. By segmenting an architecture into multiple views according to the goal interest, the authors gave a simpler view of it. Understanding the system, how to maintain it and how it develops is made easier by this description. The many architectural elements and their interrelationships are depicted in the following diagram.

**Figure 1:**
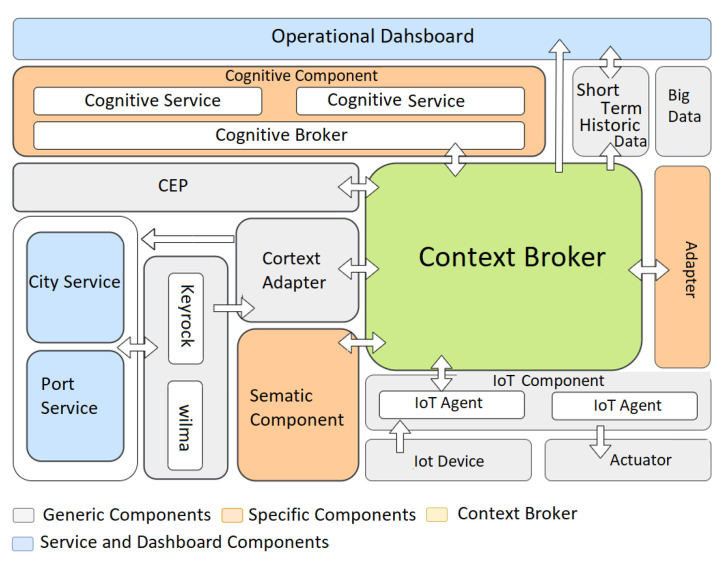
Architectural overview.

Figure 1 shows a diagram that groups the different components in colors differentiating the generic components, specific components, the context broker and the dashboards and external services.

- **Generic Components** “A platform of open source software components which can be used jointly or in combination with third-party components to build platforms that aid in the development of intelligent solutions in a fast, easy and inexpensive way” [29].
- **Specific Components** Well-defined components designed to assist the application development plan in other use cases.
- **Context Broker** A single component required to be considered a FIWARE solution.
- **External Services and Dashboard** Components responsible for creating requests to the system and displaying their responses.

For a user or a city or port service to make a request, it must first be authenticated in the system. The Idm Auth component checks if the user exists in the system. If the user does not exist, the system denies user access. Once authenticated, the user sends a request to the system. This request is evaluated by the Idm Auth component to validate if the user has permissions to perform the action. If the user is not authorized, the system denies the request. Otherwise, the Idm Auth component forwards the user’s request to the Context Adapter component.

### Summary of Useful Web Approaches

This section covers the technology required to create the web-based content creator.

The Context Adapter is the component that creates a context entity from the received request. Before sending this entity to the Context Broker component, the Context Adapter checks if there are subscriptions in the context for the type of entity to be created. To do this, it performs a query to the Context Broker component. If this does not exist, the Context Adapter creates the subscriptions and sends them to the Context Broker component. Once the existence of the subscriptions has been verified, the Context Adapter sends the entity it has created to the Context Broker component. On the other hand, this component creates and sends a response entity to the end user from the notification received from the Context Broker.

The Adapter is the component that receives a notification when an entity is created. Contextually, it corresponds to the request coming from a port service. Using the received notification, this component obtains information from the provider, required for cognitive analysis. Using this information, it creates the entity or entities in context for cognitive analysis. Before sending these entities to the context, the Adapter checks if there is a subscription in the context for each of the entities. To do so, it performs the corresponding query to the Context Broker component. If there is none, the Adapter creates the corresponding subscription and sends it to the Context Broker component. Once the existence of the subscriptions is verified, it sends the created entities to the Context Broker component.

Once the context entities required for cognitive analysis have been sent, a context entity is created corresponding to a cognitive analysis request. Again, before sending this entity to the Context Broker component, it is checked if a subscription exists in the context for this type of entity. If this does not exist, the system creates the corresponding subscription and sends it to the Context Broker component. Once the subscription is verified, it sends the entity corresponding to the cognitive analysis request to the Context Broker component.

The IoT component receives information from IoT devices and transforms it into context entities. Before sending one of these entities to the Context Broker component, the IoT component checks if there are subscriptions in the context for the type of entity to be sent. To do so, it performs the corresponding query to the Context Broker component. In case there is none, it creates a subscription for the type of entity to be sent and sends it to the Context Broker component. Once the subscriptions have been verified, it sends, to the Context Broker component, the context entity created from the information received from the IoT device. The Short Term Historic Data is the component that receives a notification from the Context Broker component each time an entity of the Adapter component and the IoT component is created in the context. From the received notification, it stores in a database the information required by the history data.

This information can be checked later by the user by visualizing it on a dashboard. The Semantic Component is the component that receives a notification from the Context Broker component each time an entity of the Adapter component and the IoT component is created in the context. It extracts the context entity included in the received notification and searches the linked database for the meaning of each entity attribute. Then, the Semantic Component adds a new metadata attribute to the entity attribute with the found meaning. Once it has added a meaning to each of the entity attributes, it sends the modified context entity back to the Context Broker component.

The Congnitive Component is the component that receives a notification from the Context Broker component each time a cognitive analysis request entity is created in the context. From the received notification, this component obtains, from its linked database, the information corresponding to the cognitive service to be executed. Once it has this information, it creates an entity that is sent to the corresponding cognitive service. The cognitive service receives an entity corresponding to a cognitive analysis request. This entity contains the identifiers of the entities required for cognitive analysis to be executed. For each of these identifiers, the corresponding context entity is obtained from the Context Broker component. Once all the necessary information is available, the cognitive analysis is performed.

As a result of the analysis, a response is created and sent. A context entity is created from this response. Before sending this entity to the Context Broker component, it is checked if a subscription for this type of entity exists in the context. If it does not exist, a subscription is created and sent to the Context Broker component. Once the subscriptions have been verified, the context entity corresponding to the result of the cognitive analysis is sent to the Context Broker component. The CEP is the component that receives a notification each time a context entity is created from the cognitive component. From the received notification, it extracts the information required by the business rule and executes the business rule. As a result of the rule execution, a context entity is created and sent to the Context Broker component, and the corresponding user or service is sent for viewing.

## 4. Machine-Learning Models for Wind-Power-Generation Prediction

In this section, we are going to study the different machine-learning models that allow prediction of the production of energy generated through windmills, using for this purpose climatological information obtained from meteorological APIs. Initially, we decided to make a previous selection of algorithms using libraries, such as pycaret, which generate a list of the algorithms that can give the best results for a previously selected data set.

As we can see in the previous image, pycaret shows us a Table 1 with different algorithms in which it has tested our data set for the previously selected characteristic, that we specifically want to predict from that data set. The algorithm with the best results was the Light Gradient Boosting Machine, which also shows us the hyperparameters it has selected for this test.

### 4.1. Study of the Dataset

Before starting to explain the machine-learning models that we finally decided to use, it is necessary to prepare the dataset with which we are going to train, validate and test our models. This process is key to achieving the expected results in our models. The dataset that was used for this project was taken from a scada system of windmills that are installed in Turkey and are generating energy [30,31].

The dataset has five columns, which are:

- **Date/Time** Time at which the measurement was taken, the measurements were taken at 10 minute intervals.
- **LV Active Power (kW)** The power generated by that mill at that time.
- **Wind Speed (m/s)** The wind speed at the height of the windmill axis.
- **Theoretical Power Curve (KW)** The theoretical power that the windmill should generate for that wind speed (provided by the manufacturer).
- **Wind Direction (°)** The wind direction at the windmill axis (The windmill is automatically rotated to that direction).

For the study of this dataset, more columns have been added [30,31]:

- **Month** A month column was added based on the date column.
- **Mean Wind Speed** In this new column, the wind speed is rewritten in 0.5 intervals—for example: if the wind speed is between 3.25 and 3.75, it becomes 3.5; and if the wind speed is between 3.75 and 4.25, it becomes 4.
- **Mean Wind Direction** In this new column, the wind direction is rewritten in 30 degree intervals—for example: for wind directions between 15 and 45, it would become 30; or for wind directions between 45 and 75, it would become 60.
- **Direction** In this column, we rewrite the Mean Wind Direction column to replace its values with letters, e.g., 0 = N, 30 = NNE, 60 = NEE and 90 = E, …

Following this, we found that most of the wind speed values were between 3.5 and 25.5, and thus we eliminated the values that are outside this range. We also observed that there were a small amount of values in which the wind speed was higher than 3.5 but the energy generated was 0—this means that the windmill is out of service, and thus they were eliminated as well.

**Figure 2:**
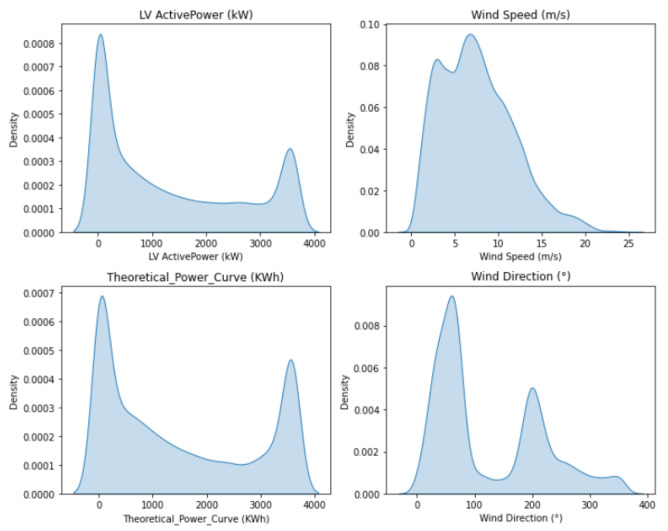
Distribution of column values.

The distribution of the values for the respective columns is shown below. The analysis used to remove wind data when the turbine is operating abnormally, such as when there is wind reduction and blade damage, will mitigate the negative effects of these abnormally high values in the training phases of the power curve model. However, it cannot be guaranteed that various types of outliers will be found and handled during the data preprocessing phase. As a result, certain hidden anomalies will still be present in the data. As a result, the distribution of errors in the modeled data for the harmonic power curve will be asymmetrical as seen in Figure 2.

**Figure 3:**
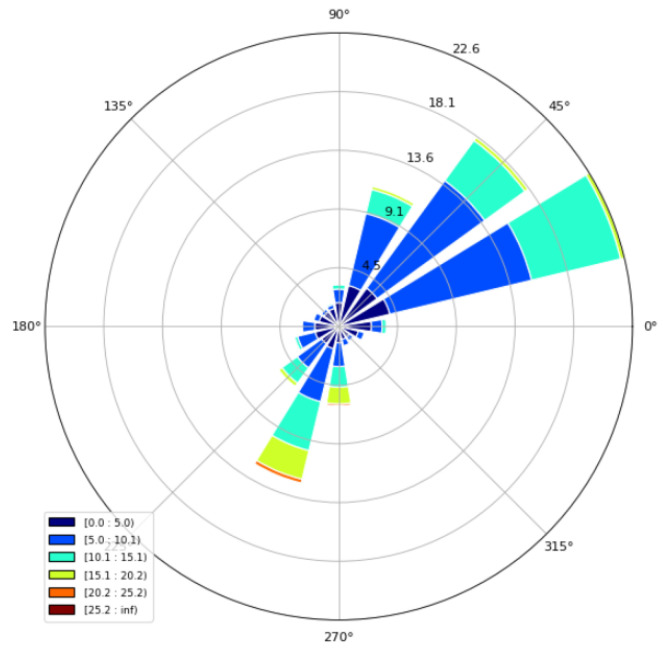
Comparison of wind speed and direction.

Below is a graph showing the wind speed and wind direction for the different measurements: The annual frequency distribution of observed wind direction and wind speed representing the relatively calm wind field at the upper boundary is seen in the wind rose in Figure 3. This analysis reveals the main flow directions in the area to be northeast, east and southwest, which is typical for the area. Therefore, the model study below focuses on the main wind directions northeast (45°), east (25°) and southwest (250°, as the 270° and 225° wind direction intervals are represented with similar frequency).

### 4.2. Algorithms Used Wind

**Figure 4:**
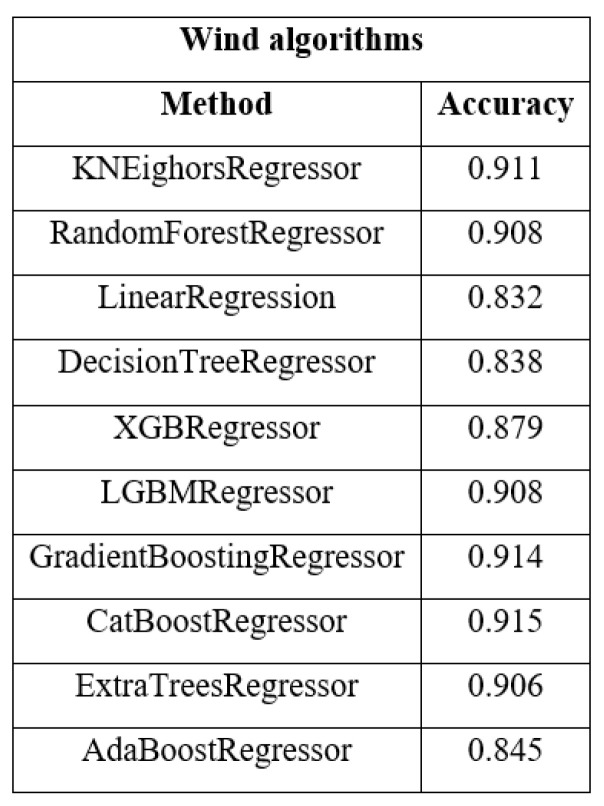
Scoring algorithms ML wind.

Once the dataset was prepared, the prediction process began. In the first instance, we tested the algorithms indicated by the pycaret library—those shown in Figure 4—with the respective parameters indicated by the library. In this way, we obtained an average score of 80–85%. In order to improve the performance of the algorithms, we selected those algorithms that exceeded 88% by retouching their hyperparameters and selected other algorithms, not indicated by pycaret, whose hyperparameters were modified, obtaining the following results.

## 5. Machine-Learning Models for Solar-Power-Generation Prediction

In this section, we study the different machine-learning models that will allow us to predict the production of energy generated through solar panels using, for this purpose, climatological information obtained from meteorological APIs. Initially, we decided to make a previous selection of algorithms using libraries, such as pycaret, which generate a list of the algorithms that can give the best results for a previously selected data set.

As we can see in the previous image, pycaret shows us a Table 2 with different algorithms in which it has tested our data set for the previously selected characteristic that we specifically want to predict from that data set. The best performing algorithm was CatBoostRegressor.

### 5.1. Study of the Dataset

Before starting to explain the machine-learning models that we finally decided to use, it is necessary to prepare the dataset with which we are going to train, validate and test our models. This process is key to achieve the expected results in our models.

The dataset used for this project was obtained from a solar panel farm in Berkeley, CA [32].

The dataset has 16 columns, which are as follows:

- **Day of Year** Day of current year [0–365].
- **Year** Year in which the measurement is taken.
- **Month** Month of the year in which the measurement is taken.
- **Day** Day of the month in which the measurement is taken.
- **First Hour of Period** Measurements are taken in 3 h intervals, i.e., each measurement represents the value of a 3 h interval, and this column represents the time of day when that 3 h period begins.
- **Daylight** Represents whether the time that measurement was taken was in the daytime or nighttime.
- **Distance to Solar Noon** A measure representing the distance to the time of day when the sun is at the highest point in the sky for the location where the measurements are taken [0–1].
- **Average Temperature (Day)** Average of the temperature of the day when the measurement is taken.
- **Wind Direction (Day)** Average wind direction for the day the measurement is taken.
- **Average Wind Speed (Day)** Average wind speed for the day the measurement is taken.
- **Sky Cover** Indicates how clear the sky is.
- **Visibility** Indicates the visibility.
- **Relative Humidity** Relative humidity of the environment.
- **Wind Speed (Period)** Average wind speed of the period in which the measurement is taken.
- **Barometric Pressure (Period)** Average barometric pressure of the period in which the measurement is taken.
- **Generated** Amount of power generated in that period.

**Figure 5:**
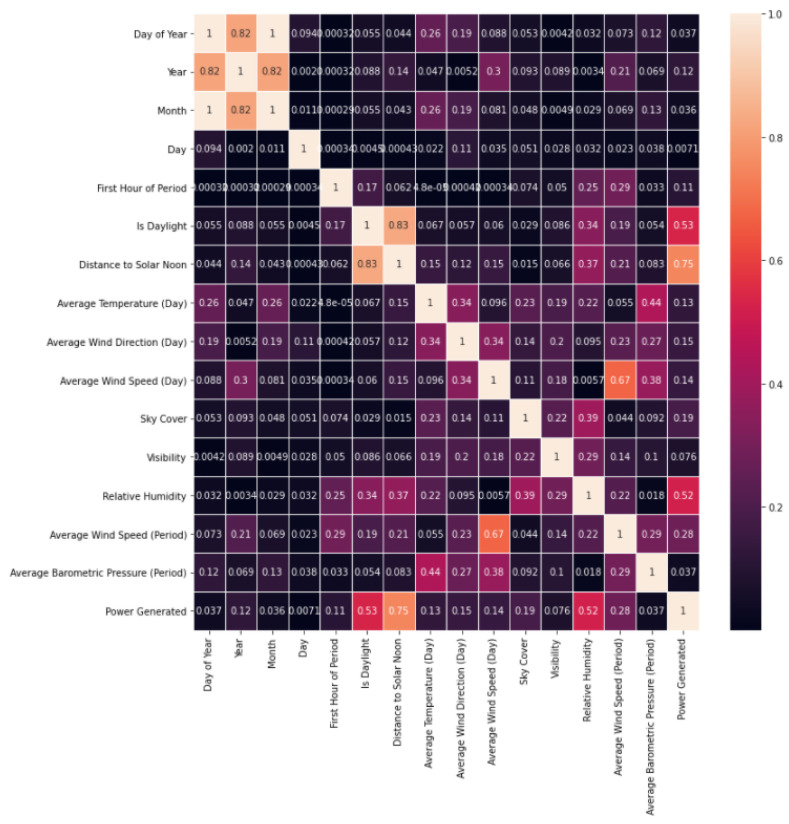
Relationship between columns of the dataset.

We observe the relationship between the different columns of the dataset through a heat map Figure 5.

### 5.2. Algorithms Used Solar

**Figure 6:**
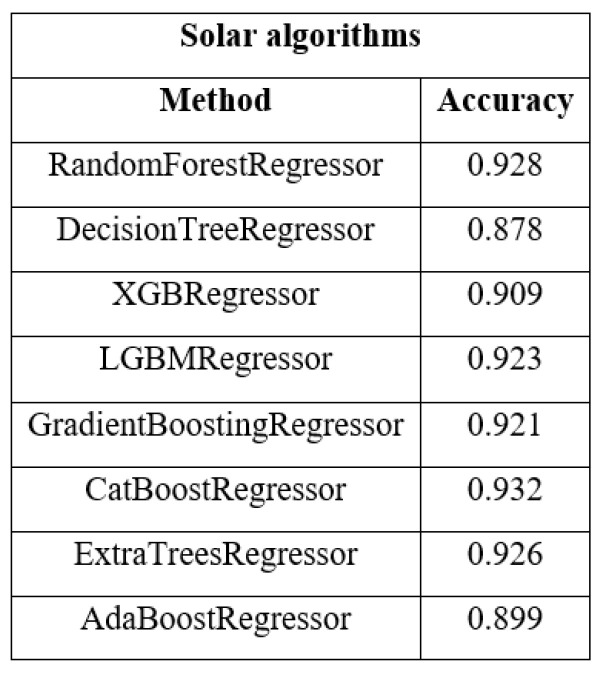
Scoring algorithms ml solar.

Once the dataset was prepared, the prediction process began. In the first instance, we tested the algorithms indicated by the pycaret library—those shown in Figure 6—with the respective parameters indicated by the library. In this way, we obtained an average score of 83–87%, in order to improve the performance of the algorithms, we selected those algorithms that exceeded 88% by retouching their hyperparameters and selected other algorithms, not indicated by pycaret, whose hyperparameters were modified, obtaining the following results:

## 6. Trading

As for the negotiation part, an algorithm based on the sealed envelope auction was developed. In this auction, bidders put the price of the auctioned object in an envelope, and the one who has written the highest price wins the auction. For adaptation to the digital format, features have been added for the benefit and convenience of users to facilitate the process of bidding and selling. In the following, the process of putting up for sale and auctioning the different energy lots is detailed. First, a producer divides its power generation forecast into lots. These lots are automatically assigned the initial bid price and the maximum price that they are able to reach on the market.

These prices are set by Red Eléctrica de España and provided by eSios through its API. However, the sale prices to small electricity consumers are set after 8:00 PM for the following day, which limits energy trading between 8:00 PM and 12:00 PM. Faced with this drawback, a trading process capable of operating in that time interval had to be created. A system that would force a multitude of users to participate simultaneously in several auctions in which they were interested in such a short period of time each day would not be successful.

Therefore, it was determined that the sealed bid auction provided more advantages than the traditional English auction, having to place a single bid. However, setting a single bid for all the energy lots in which the user would be interested would greatly limit the benefit that could be obtained, either by setting the price too high or too low. To address the regret factor present in sealed bid auctions, the blind bid was adapted to be treated as a maximum price willing to bid per lot if necessary. Although this did not completely solve the problem, it did serve to alleviate the effect of regret.

With this, it is possible to automate the bidding process to a certain extent, reducing the interaction required by the user. In this negotiation process, the user is given the freedom to participate in all the auctions in which they can supply the energy need. This means that the consumer can participate in all auctions whose energy lot provides an amount less than or equal to their energy requirements. This avoids wasting energy on much lower requirements with lots of high energy input.

Following pre-negotiation, the auction process of an energy lot begins. First, it is verified that the users meet the necessary requirements to participate in the lot auction, these requirements being an energy requirement lower or equal to the energy supply auctioned and a closed bid equal to or higher than the initial bid of the lot. After verification, the negotiation continues. In the next stage, three situations may occur:

1. No bidders for the auction: the auction fails and no one wins the lot.
2. Single bidder for the auction: The consumer wins the auction with the starting bid price.
3. Multiple bidders for the auction: The winner selection process begins.

In the third situation, if there is only one bidder with the maximum bid, this consumer wins the auction with a bid price one unit higher than the second highest bid. In this way, we partially mitigate the regret factor and achieve a greater benefit for the consumer. In the event that there are two or more bidders with the highest bid, we proceed to a random winner selection process among the highest bidders. The resulting bidder wins the auction with the price of their sealed bid.

After winning an auction, the energy contributed from the lot is subtracted from the winner’s energy requirement, so that they can continue to participate in the remaining auctions until their needs are met. The auction result is then sent to the Orion Context Broker for processing and storage.

## 7. Discussion of Future Research Directions

The energy industry is on the verge of a true internet revolution. It intends to bring a new era of web interaction through the adoption of the Semantic Web, with significant changes in the way developers and content creators use it. This web will make web services, applications and power exchange agents more intelligent and even provide new autonomous forms of rapid response to random changes that, under human conditions, would be unresponsive through the use of an AI system. Despite the tremendous amount of innovation, its adoption in the Smart City may bring considerable challenges.

The problem with "web semantics" is that it requires a level of implementation commitment from web developers and content creators that will not be easy to achieve. In order for the semantic web developer community to help contribute to future energy development, it is essential to point them in the right direction. The following are the main challenges facing Semantic Web Development in general: content accessibility, ontological expansion, scalability, multilingualism and visualization, of which the vast majority are addressed in this research.

One of the greatest challenges associated with the adoption of the Semantic Web is the vulnerability of connected data. In the course of the research, this difficulty was discovered to exist, and future research efforts are proposed. All of a user’s personal information and token exchange records are stored and connected at one point, and a malicious party could take control of these records by corrupting the data and compromising the functionality of the system.

In addition, we intend to work with more semantic methods, such as GeoSPARQL and temporal RDF, as well as a wider range of energy-related datasets, such as remote sensing data, sensing data, energy markets and political data, with the goal of developing a robust and interoperable ontology model capable of serving a nearly complete knowledge-sharing energy data ecosystem spanning multiple domains, thereby, improving the understanding of the decentralized energy distribution mechanism as well as investigating the use of blockchain technology for peer-to-peer energy markets given that it has demonstrated a significant potential for acceptance in the P2P energy market with an increasing number of businesses adopting the technology and changing their business models [33,34].

## 8. Conclusions

COSIBAS seeks to develop an intelligent solution across multiple sectors based on open standards and open-source paradigms that provides process automation across the value chain and easy integration with other applications and services. Ensuring interoperability between heterogeneous IoT systems by defining a unified vocabulary to be shared between IoT devices and systems, based on contextual information management services and Big Data in the cloud, this study detailed and analyzed semantic-based approaches for IoT-domain representation, context management, data sharing and the definition of harmonized data models.

This last aspect is crucial, proof of which is that the API has been adopted as the first open license API standard, intended to provide the basic artifact for portability and interoperability in smart cities. The AI and ML platforms discussed in this paper provide a cloud infrastructure through which they offer a variety of services for AI/ML with algorithms that are already trained for certain functionalities within a proprietary solution. Therefore, this limits both their extensibility and their interoperability with other platforms to external providers.

On the other hand, the use of the infrastructure provided by these platforms implies a cost for the user that varies according to the usage, volume and versions of the different products/services offered. COSIBAS allows developers to add functionality aimed at providing cognitive capabilities without losing the main features of modularity, flexibility, extensibility, interoperability, standardization and free open source, making this IoT platform one of the leading ones in the market.

## Figures and Tables

**Table 1:** Pycaret pre-study.

Model	MAE	MSE	RMSE	R^2^	RMSLE	TT (s)
Light Gradient Boosting	152.6337	141,232.3644	375.287	0.918	1.508	0.127
Gradient Boosting	155.4339	141,346.4811	375.4099	0.9179	1.6532	0.94
CatBoost	154.6182	143,318.3611	378.0807	0.9168	1.6128	2.963
Extreme Gradient Boosting	157.3401	148,772.8508	385.1605	0.9136	1.5959	1.129
Random Forest	160.9863	152,788.12 10	390.4444	0.9113	1.3707	2.217
Extra Trees	166.4859	158,063.3209	397.1247	0.9082	1.3746	1.07
K Neighbors Regressor	171.5040	165,489.9156	406.3238	0.9039	1.3991	0.024
Ada Boost	309.1532	246,932.7357	496.5982	0.8566	2.0464	0.088
Decision Tree	199.8973	277,651.8974	526.6256	0.8387	1.8514	0.049
Linear Regression	385.0205	283,773.3672	532.5063	0.8352	2.6316	0.425
Bayesian Ridge	385.0236	283,773.3669	532.5063	0.8352	2.6316	0.007
Ridge	385.0258	283,773.3562	532.5063	0.8352	2.6315	0.006
Least Angle	385.0206	283,773.3667	532.5063	0.8352	2.6316	0.008
Lasso	385.1961	283,776.5953	532.5101	0.8352	2.6312	0.007
Orthogonal Matching Pursuit	385.9314	284,452.3678	533.1459	0.8348	2.6404	0.007
Huber Regressor	378.314 1	295,961.1937	543.7595	0.8281	2.7125	0.032
Passive Agg ressive	378.0183	303,116.3767	550.2841	0.8239	2.768	0.014
Lasso LeastAngle	429.8264	316,211.4787	562.2203	0.8163	2.5564	0.016
Elastic Net	547.8478	444,773.0906	666.8626	0.7415	2.8225	0.007

**Table 2:** Initial study of pycaret.

Model	MAE	MSE	RMSE	R^2^	RMSLE	MAPE
CatBoost	1383.53	8,258,035.897	2842.3398	0.92	3.368	1.4268
Light Gradient Boosting Machine	1425.90	8,645,953.306	2917.0007	0.9167	3.1948	1.444
Gradient Boosting	1525.33	9,042,987.79	2973.3297	0.9129	3.4837	1.9477
Random Forest	1387.55	9,132,938.701	2988.8308	0.9122	1.4369	1.3502
Extreme Gradient Boosting	1528.08	9,132,115.59	2989.7954	0.912	3.5136	1.9225
Extra Trees	1371.164	9,370,078.345	3032.71	0.9091	1.4207	1.2388
K Neighbors	1995.01	14,418,032.69	3783.2075	0.8593	1.8795	3.2709
AdaBoost	2658.09	16,246,782.77	4011.6881	0.8427	4.5131	3.6487
Decision Tree	1823.12	18,487,851.69	4231.6982	0.8231	1.7356	1.2967
Linear	3320.22	21,609,347.74	4633.5979	0.7902	5.0483	6.1362
Lasso	3321.05	21,609,322.51	4633.5942	0.7902	5.0499	6.1158
Bayesian Ridge	3328.44	21,619,397.04	4634.7018	0.7901	5.0601	6.103
Ridge Regression	3330.88	21,622,794.72	4635.0831	0.79	5.0622	6.0973
Lasso Least Angle	3341.40	21,757,851.6	4649.9118	0.7888	5.0506	5.7934
Random Sample Consensus	3260.63	21,838,823.48	4657.6009	0.7878	5.0006	6.0694
TheilSen Regressor	3436.71	22,350,259.85	4713.5883	0.7825	5.1132	6.0852
Huber Regressor	3054.06	23,610,757.4	4837.1228	0.7706	4.615	4.3961
Passive Aggressive	3018.56	26,104,737.57	5088.1992	0.747	4.1218	3.0214
Elastic Net	4701.35	37,866,615.25	6143.6763	0.6359	5.2276	8.0117
Orthogonal Matching Pursuit	4881.56	38,453,549.95	6193.5956	0.6299	5.3537	5.5916
Least Angle Regression	5456.26	63,265,660.21	7134.0762	0.4037	5.4275	10.456
Support Vector Machine	6928.34	147,606,069.7	12,099.8444	-0.3985	4.5004	1.4865

## Data Availability

Not applicable.
